# Genome-wide analysis of soybean cinnamyl alcohol dehydrogenase genes identifies GmCAD3 as a positive regulator of *Fusarium oxysporum* resistance

**DOI:** 10.3389/fpls.2025.1731612

**Published:** 2025-12-11

**Authors:** Runnan Zhou, Jia You, Jinrong Li, Han Li, Sobhi F. Lamlom, Honglei Ren, Jiajun Wang

**Affiliations:** 1 Soybean Research Institute of Heilongjiang Academy of Agriculture Sciences, Harbin, China; 2 Plant Production Department, Faculty of Agriculture Saba Basha, Alexandria University, Alexandria, Egypt

**Keywords:** soybean, cinnamyl alcohol dehydrogenase, *Fusarium oxysporum*, gene function characterization, haplotype analysis

## Abstract

**Background:**

Cinnamyl alcohol dehydrogenase (CAD) catalyzes the final step in monolignol biosynthesis and plays critical roles in lignin-mediated plant defense against pathogens. Despite soybean global agricultural importance, systematic characterization of the CAD gene family and its role in disease resistance remains lacking.

**Methods:**

We performed comprehensive genome-wide identification, phylogenetic analysis, and expression profiling of soybean CAD genes. Haplotype diversity was assessed through whole-genome resequencing of 289 Chinese soybean accessions. Population genetic parameters (Fst, π, Tajima’s D) were calculated to examine selection signatures. Functional validation was conducted through promoter activity assays, subcellular localization, and transgenic hairy root overexpression challenged with *Fusarium oxysporum*.

**Results:**

Seven *GmCAD* genes with conserved catalytic domains (PF00107, PF08240) were identified and grouped into four phylogenetic clusters. Expression profiling revealed strong pathogen-induced upregulation, with *GmCAD3* showing the most dramatic response (8.64-fold induction at 14 days post-inoculation). Three major *GmCAD3* haplotypes were identified based on seven SNPs (five promoter, two coding sequence). Haplotype 2 exhibited significantly enhanced disease resistance (disease severity index 72.4 ± 2.1) compared to Haplotype 1 (88.6 ± 1.8; *P* < 0.001), correlating with superior promoter activity. Population genetic analysis indicated maintenance of multiple functional haplotypes during domestication. Transgenic validation confirmed that *GmCAD3* overexpression increases CAD enzyme activity (2–3 fold), lignin accumulation (70-88%), and pathogen resistance.

**Conclusions:**

This study provides the first comprehensive characterization of the soybean CAD gene family and demonstrates that *GmCAD3* functions as a key regulator of lignin-mediated defense against *F. oxysporum*. Natural haplotype variation in *GmCAD3* provides valuable molecular markers for breeding *Fusarium*-resistant soybean cultivars.

## 1 Introduction

Soybean (*Glycine max L*. Merr.) is one of the most economically important legume crops worldwide, providing essential protein and oil resources for human consumption and animal feed (Lamlom et al., 2020; Ren et al., 2023). With global demand continuously rising, maximizing soybean productivity has become a critical priority for food security (Cheng et al., 2025). However, soil-borne diseases, particularly those caused by fungal pathogens, pose significant threats to soybean production, causing substantial yield losses and quality deterioration (Tripathi et al., 2022). Among these pathogens, *Fusarium oxysporum* is a devastating soil-borne fungus that causes Fusarium root rot (FRR), leading to root necrosis, vascular browning, leaf chlorosis, and plant wilting (Zhao et al., 2024; Liu et al., 2025c). Traditional disease management strategies relying on chemical fungicides are becoming increasingly problematic due to environmental concerns, rising costs, and the emergence of resistant pathogen strains. Consequently, developing disease-resistant cultivars through genetic improvement represents the most sustainable and practical approach to combat FRR in soybean production systems (Mahmoud, 2021).

Plant defense mechanisms against pathogen invasion involve complex biochemical and structural modifications, with cell wall reinforcement through lignin deposition playing a pivotal role (Gao et al., 2024; Uddin et al., 2024). Lignin, a complex phenolic polymer, is synthesized from three primary monolignols: p-coumaryl alcohol, coniferyl alcohol, and sinapyl alcohol, which form hydroxyphenyl (H), guaiacyl (G), and syringyl (S) units, respectively (Zhou et al., 2025a). The accumulation of lignin in cell walls creates physical barriers that restrict pathogen penetration and colonization, thereby conferring disease resistance (Sattler and Funnell-Harris, 2013). Recent studies have demonstrated the importance of lignin biosynthesis in plant immunity. For example, overexpression of *AcLac35* enhances lignification and resistance to Pseudomonas syringae pv. actinidiae in kiwifruit (Li et al., 2025), while the rice transcription factor bHLH25 coordinately regulates lignin biosynthesis and antimicrobial compound production to confer broad-spectrum disease resistance (Liao et al., 2025).

CAD is a key regulatory enzyme in the lignin biosynthetic pathway, catalyzing the final step of monolignol biosynthesis by reducing cinnamaldehydes to their corresponding cinnamyl alcohols (Sibout et al., 2005; Zhang et al., 2022). As members of the medium-chain dehydrogenase/reductase (MDR) superfamily, CAD enzymes possess conserved catalytic domains that are essential for their enzymatic function (Ma et al., 2024; Wang et al., 2024). The CAD gene family has been extensively characterized in several plant species, revealing diverse roles in development and stress responses. In Arabidopsis thaliana, nine AtCAD genes have been identified, with AtCAD4 and AtCAD5 playing predominant roles in lignin synthesis in reproductive tissues and stems (Kim et al., 2004; Liu et al., 2025a, b). Cotton *GhCAD7* specifically regulates syringyl lignin biosynthesis, affecting lignin composition and fiber quality (Zhang et al., 2020; Li et al., 2022). Notably, wheat *TaCAD12* has been shown to contribute to disease resistance against sharp eyespot by upregulating defense-related genes and modulating monolignol biosynthesis (Rong et al., 2016). These studies highlight the multifunctional roles of CAD genes in both developmental processes and pathogen defense. However, our understanding of soybean *CAD* genes and their roles in lignin biosynthesis and FRR resistance remains limited.

In this study, we performed a comprehensive genome-wide identification and characterization of the CAD gene family in soybean. We systematically analyzed their phylogenetic relationships, conserved protein domains, gene structures, cis-regulatory elements, and tissue-specific expression patterns. Through expression profiling under Fusarium oxysporum infection, we identified GmCAD3 as a highly responsive gene to pathogen challenge. We further investigated the natural variation of GmCAD3 across 289 soybean germplasm accessions, identifying three major haplotypes with differential disease resistance phenotypes. Functional validation through promoter activity analysis, subcellular localization, and transgenic hairy root assays demonstrated that GmCAD3 enhances disease resistance by increasing CAD enzyme activity and lignin accumulation. Population genetic analysis revealed signatures of balancing selection, indicating that GmCAD3 haplotype diversity has been maintained during domestication. Our findings provide fundamental insights into the role of CAD genes in soybean immunity and offer valuable molecular markers for breeding programs aimed at developing Fusarium-resistant soybean varieties.

## 2 Materials and methods

### 2.1 Identification and characterization of CAD genes in soybean

Soybean protein datasets and complete genome sequences were obtained from the Phytozome v12.1 database (https://phytozome.jgi.doe.gov/pz/portal.html). To identify putative CAD family members, we used characterized CAD protein sequences from Arabidopsis thaliana, retrieved from The Arabidopsis Information Resource (TAIR; https://www.arabidopsis.org/), as query sequences for BLASTP searches against the soybean proteome. An initial BLASTP search was performed using an E-value cutoff of 1.0 to maximize sensitivity in candidate retrieval. Putative CAD sequences were then verified using InterProScan and SMART to assess specificity. All candidate sequences were validated for the presence of characteristic CAD domains (PF00107 and PF08240) using InterProScan (version 5.52-86.0) with default parameters and SMART databases (version 9.0). Only sequences with E-values < 0.01 for both domains and covering at least 80% of the domain length were retained (Ma et al., 2018). Duplicate entries were manually eliminated from the dataset. Physicochemical properties including protein length, molecular mass, and theoretical pI values were determined using the ExPASy proteomics server (https://www.expasy.org/) (Gasteiger et al., 2005). Predicted subcellular distribution of *GmCAD* proteins was determined using WoLF PSORT (https://www.genscript.com/wolf-psort.html).

### 2.2 Evolutionary and structural analysis

For phylogenetic reconstruction, CAD amino acid sequences from six plant species *Arabidopsis thaliana, Glycine max, Zea mays, Oryza sativa, Phaseolus vulgaris*, and *Sorghum bicolor* were collected (**Supplementary Table S1**). Sequence alignment was performed using ClustalW, followed by phylogenetic tree construction in MEGA 5.0 with the neighbor-joining method (Tamura et al., 2011). Domain conservation was verified through sequence analysis. The Gene Structure Display Server v2.0 was used to visualize the intron-exon organization of soybean CAD genes. Syntenic relationships between CAD genes from the six species were analyzed using the Plant Genome Duplication Database (PGDD, https://chibba.agtec.uga.edu/duplication/).

### 2.3 Promoter Cis-element identification

Upstream regulatory sequences (2000 bp) of each GmCAD gene were extracted and submitted to PlantCARE (https://bioinformatics.psb.ugent.be/webtools/plantcare/html/) for cis-acting element prediction (Liu et al., 2015).

### 2.4 Transcriptional profiling of *GmCADs*

Expression data for *GmCADs* in different tissues (leaves, roots, seeds, pods, flowers) were retrieved from RNA-seq datasets available in the Phytozome database. Heatmap visualization was performed using TBtools software (Chen et al., 2020). For pathogen challenge experiments, the susceptible cultivar ‘DN50’ was inoculated using a sorghum-based inoculation system adapted from Trivedi et al (Trivedi et al., 2023). *Fusarium oxysporum* was propagated on potato dextrose agar (PDA) at 26°C in darkness for 7 days. The culture was then transferred onto autoclaved sorghum grains and incubated under identical conditions for 17 days until complete colonization. Plastic pots (11 cm height) were filled two-thirds full with sterilized vermiculite, and colonized sorghum grains were incorporated at 4% (w/w). After adding a 0.5 cm vermiculite layer, 15 soybean seeds were planted per pot and wholly covered with sterile vermiculite. Three biological replicates were established for each treatment. Plants were grown in controlled-environment chambers at 25°C under a 14 h light/10 h dark cycle. Root tissue was harvested at 8, 11, 14, and 17 days after inoculation, immediately frozen in liquid nitrogen, and stored at -80°C. Total RNA extraction was performed using the Quick Total RNA Isolation Kit (Huayueyang Biotechnology, Beijing). RNA integrity and concentration were assessed using a Nanodrop2000 spectrophotometer. First-strand cDNA synthesis was carried out using PrimeScript^®^ RT Master Mix (TaKaRa, Dalian) with 500 ng total RNA as template, followed by dilution to 500 ng/μL. Quantitative RT-PCR reactions were conducted on a Roche LightCycler^®^ 96 platform using SYBR Green chemistry (TaKaRa). Each 20 μL reaction mixture contained 10 μL of 2× SYBR Green Master Mix, 0.5 μL of each primer (10 μM), and appropriate cDNA template. Thermal cycling parameters were: 95°C for 30 s, followed by 40 cycles of 95°C for 10 s and 60°C for 30 s. *GmACTIN* (GenBank: AF049106) served as the internal reference gene. Relative transcript abundance was calculated using the 2^ΔΔCt^ method. ΔΔCt = (Ct target gene - Ct reference gene) treatment - (Ct target gene - Ct reference gene)control. The control group (CK, non-inoculated plants) at each time point served as the calibrator sample. Primer sequences are provided in **Supplementary Table S1** (Sang et al., 2023).

### 2.5 Haplotype diversity analysis of *GmCAD3*

*GmCAD3* haplotype variation was examined across 289 Chinese soybean accessions using unpublished whole-genome resequencing data. The reference *GmCAD3* sequence was obtained from Phytozome and aligned against resequencing data to detect single-nucleotide polymorphisms (SNPs). DnaSP 5.0 was used to analyze haplotype structure, with low-frequency haplotypes (<5%) excluded (Ruihong et al., 2006). Statistical comparisons of phenotypic differences between haplotypes were performed using one-way ANOVA followed by Duncan’s *post-hoc* test in GraphPad Prism 9.5.1.

### 2.6 Selection signature analysis

To evaluate selective pressures on *GmCAD3* during soybean domestication, we analyzed publicly available genomic data from 2,790 accessions in the SoyMD database (https://yanglab.hzau.edu.cn/SoyMD/#/). Population genetic parameters, including fixation index (Fst), nucleotide diversity (π), and Tajima’s D, were calculated for GmCAD3 regions. The dataset included 591 wild soybean accessions, 439 Huang-Huai-Hai landraces, 379 Huang-Huai-Hai cultivars, 539 northern landraces, and 842 northern cultivars (Yang et al., 2023).

### 2.7 Subcellular localization

Complete coding sequences of *GmCAD3* haplotypes were amplified from roots of representative soybean varieties (Hap1/3 and Hap2). These sequences were fused to GFP at the C-terminus and cloned into pBI121 vectors through homologous recombination. Arabidopsis thaliana mesophyll protoplasts were isolated and transformed via PEG-calcium-mediated transfection, with a 16-hour incubation period (Yoo et al., 2007). An empty GFP vector served as control. Fluorescence imaging was conducted using a Carl Zeiss LSM710 confocal microscope. *AtHSBP*-RFP was used as a cytoplasmic reference marker (Hsu et al., 2010).

### 2.8 *GmCAD3* promoter activity analysis

Promoter regions (2000 bp upstream of start codon) from each *GmCAD3* haplotype were amplified and inserted upstream of the luciferase reporter gene in CP461 vectors via homologous recombination, generating proGmCAD3-Hap1::Luc, proGmCAD3-Hap2::Luc, and proGmCAD3-Hap3::Luc constructs. These plasmids were introduced into Agrobacterium tumefaciens EHA105 (Weidi, Shanghai) by chemical transformation. Bacterial suspensions were infiltrated into Nicotiana benthamiana leaves. At 48 hours post-infiltration, leaves were sprayed with Luciferin substrate (Promega, USA) and incubated 5–10 minutes before imaging with a Tanon system (Tanon, China). For quantitative measurements, infiltrated leaf tissue was collected at 48 hpi, ground in liquid nitrogen, and lysed. Luminescence was quantified using a dual-luciferase reporter assay system (Promega, USA) following manufacturer guidelines. Firefly luciferase (LUC) signals were measured first, then Renilla luciferase (REN) signals after LUC quenching (Zhou et al., 2025b).

### 2.9 Hairy root transformation

Coding sequences of *GmCAD3* haplotypes (Hap1/3 and Hap2) were cloned into *pCAMBIA3301* under CaMV 35S promoter control, creating *pCAMBIA3301-GmCAD3*-Hap1/3 and *pCAMBIA3301-GmCAD3*-Hap2. These constructs were transformed into Agrobacterium rhizogenes K599 and used to infect hypocotyls of ‘DN50’ seedlings. Hairy root induction and selection followed established protocols (Zhou et al., 2025c). Non-infected roots served as negative controls. QRT-PCR and fluorescence microscopy confirmed the presence of transgenic roots, and non-transgenic roots were removed. At 17 days post-pathogen inoculation, disease severity, root length, and fresh weight were measured in both control hairy roots (CHR) and overexpression hairy roots (OHR).

### 2.10 Enzyme activity and lignin quantification

At 14 days after *Fusarium oxysporum* inoculation, CAD enzyme activity and total lignin content were determined in wild-type and *GmCAD3*-overexpressing hairy roots using commercial kits (CAD Activity Assay Kit, AKSU053U; Lignin Content Assay Kit, AKSU010U; BOXBIO, China). For each assay, 0.1 g of root tissue was processed according to kit protocols.

### 2.11 Data analysis

Statistical analyses were conducted using SPSS 25 and GraphPad Prism 9.5.1. Data normality was assessed using Shapiro-Wilk tests, and homogeneity of variance was evaluated using Levene’s test. For pairwise comparisons, Student’s t-tests were performed. For multiple group comparisons, one-way ANOVA followed by Duncan’s *post-hoc* test was used. For haplotype disease severity index (DSI) analysis, one-way ANOVA with Tukey’s HSD *post-hoc* test was applied. Significance threshold was set at P < 0.05. For multiple comparisons in expression profiling across tissues and timepoints, false discovery rate (FDR) correction was applied using the Benjamini-Hochberg method.

## 3 Results

### 3.1 Identification and sequence analysis of *CAD* family genes in soybeans

To identify members of the *GmCAD* gene family, the known CAD protein sequences from Arabidopsis thaliana were compared against the Phytozome v12.1 database. As summarized in **Table 1** and **Supplementary Table S2**, a total of seven cytosolic *GmCAD* genes (*GmCAD1-7*) were identified in the soybean genome, with ORF lengths ranging from 870 bp (*GmCAD5*) to 1095 bp (*GmCAD3*). These genes encode proteins of 289–364 amino acids, with predicted molecular weights of 31.43–39.37 kDa and theoretical isoelectric points (pI) between 5.51 and 6.86 (**Table 1**).

**Table 1:** Basic characteristics of the seven *GmCAD* genes identified in soybean.

Gene Name	Gene ID	Gene location	ORF length(bp)	Protein length(aa)	Isoelectric point	Molecular weight (KDa)	Subcellular localization
*GmCAD1*	*Glyma.01G020900*	Gm01:2111576-2116367	1089	362	6.37	39.13	Cytoplasm
*GmCAD2*	*Glyma.01G021000*	Gm01:2129327-2132211	1080	359	6.37	38.94	Cytoplasm
*GmCAD3*	*Glyma.09G201200*	Gm09:43427921-43430353	1095	364	5.92	39.04	Cytoplasm
*GmCAD4*	*Glyma.10G262400*	Gm10:48777272-48780935	1077	359	5.61	39.15	Cytoplasm
*GmCAD5*	*Glyma.16G096300*	Gm16:18446114-18447221	870	289	5.91	31.43	Cytoplasm
*GmCAD6*	*Glyma.18G177000*	Gm18:42493460-42499284	1086	361	6.86	39.37	Cytoplasm
*GmCAD7*	*Glyma.20G128600*	Gm20:37000973-37002958	1074	357	5.51	39.03	Cytoplasm

**Figure 1:**
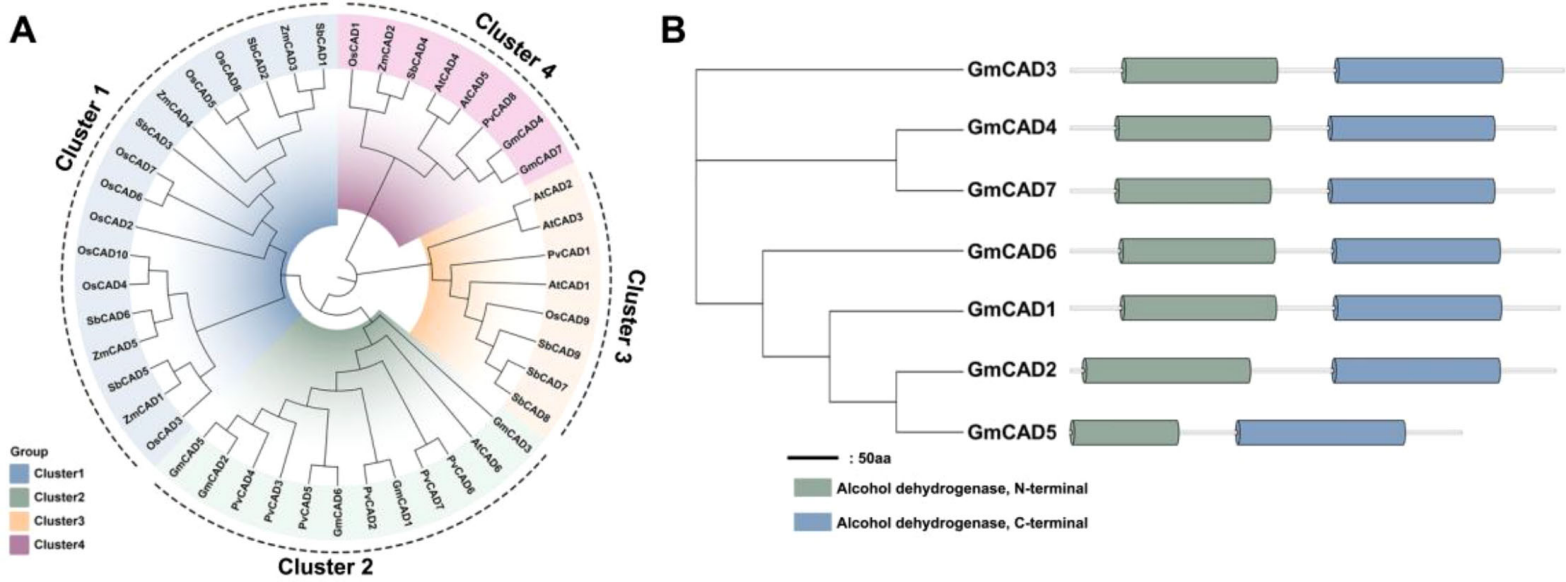
Phylogenetic relationships and conserved domain structures of CAD proteins in soybean and other plant species. **(A)** Phylogenetic analysis of CAD proteins from multiple species (*Arabidopsis thaliana*, *Glycine max*, *Zea mays*, *Oryza sativa*, *Phaseolus vulgaris*, and *Sorghum bicolor*). The phylogenetic tree was constructed using the neighbor-joining method with 1,000 bootstrap replicates. CAD proteins are grouped into four distinct clusters (I-IV) based on evolutionary relationships. **(B)** Conserved domain architecture of GmCAD proteins. All seven GmCADs contain two characteristic domains: PF08240 (alcohol dehydrogenase GroES-like domain, N-terminal, orange/yellow boxes) and PF00107 (zinc-binding dehydrogenase domain, C-terminal, blue/green boxes). Numbers above each domain indicate amino acid positions (start-end). The horizontal scale bar represents protein length in amino acids (aa). Detailed domain coordinates and E-values are provided in **Supplementary Table S3**.

To elucidate the evolutionary relationships of *CAD* genes, a phylogenetic tree was constructed using 45 CAD proteins from six species: rice (10), soybean (7), *Arabidopsis thaliana* (6), maize (5), common bean (8), and sorghum (9). The CAD proteins were classified into four distinct clusters based on their phylogenetic relationships (**Figure 1A**). Cluster 1 primarily contained four sorghum, four maize, and eight rice CAD members. Cluster 2 was mainly composed of five soybean members (*GmCAD1*, *GmCAD2*, *GmCAD3*, *GmCAD5*, *GmCAD6*), six common bean members, and *AtCAD6* from *Arabidopsis thaliana*. Cluster 3 largely comprised three sorghum members, three *Arabidopsis thaliana* members, the common bean member *PvCAD1*, and the rice member *OsCAD9*. Cluster 4 contained two soybean members (*GmCAD4* and *GmCAD7*), two *Arabidopsis thaliana* members, and one member each from rice, maize, sorghum, and common bean. Domain analysis revealed that the CAD proteins contain two conserved domains (PF08240, alcohol dehydrogenase N-terminal; PF00107, alcohol dehydrogenase C-terminal), consistent with previously reported domains and responsible for CAD catalytic activity (**Figure 1B**).

### 3.2 Syntenic relationships and tissue-specific expression analysis of *GmCADs*

**Figure 2:**
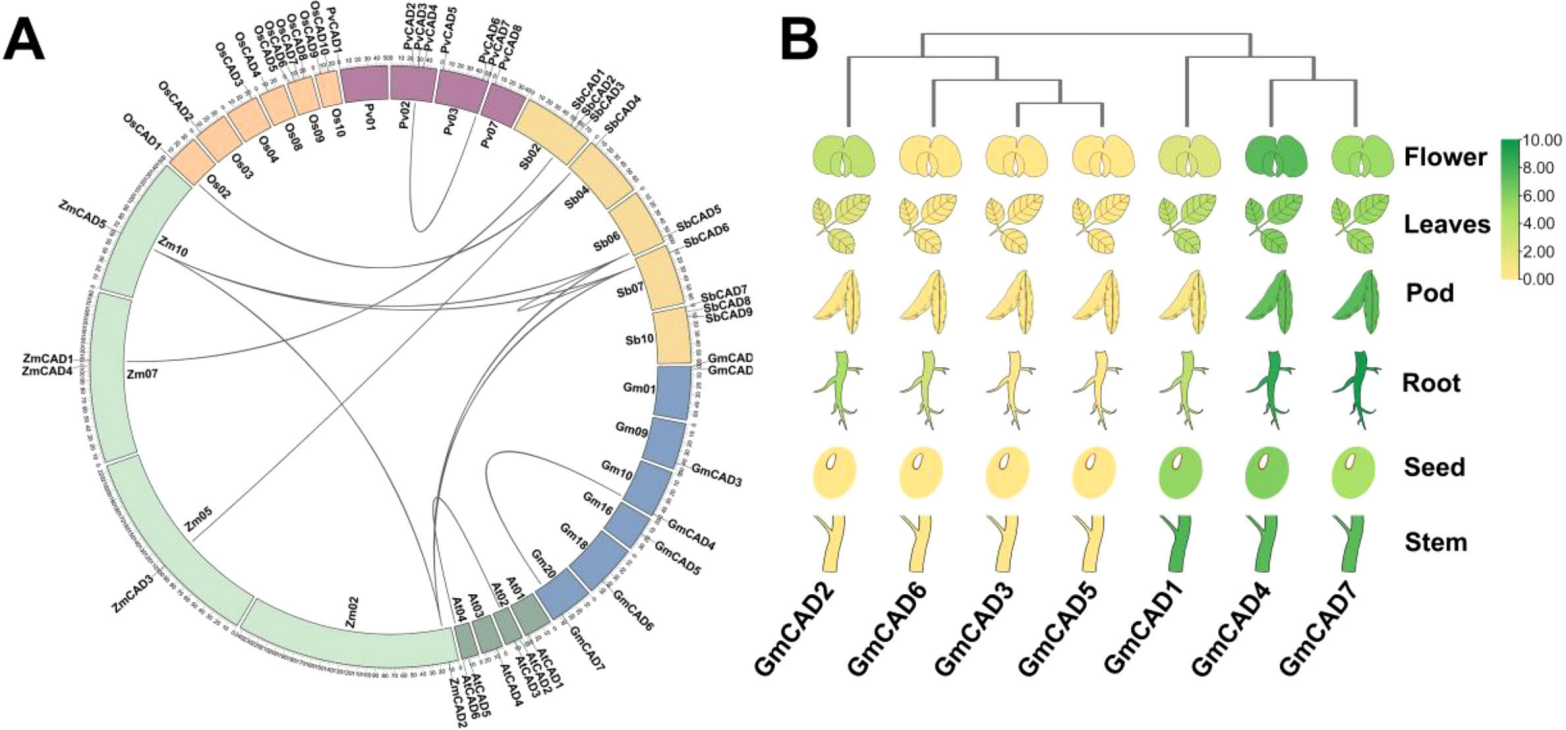
Synteny and tissue-specific expression profiling of *GmCADs*. **(A)** Syntenic analysis of *CAD* genes from *Arabidopsis thaliana* (At), *Glycine max* (Gm), *Zea mays* (Zm), *Oryza sativa* (Os), *Phaseolus vulgaris* (Pv), and *Sorghum bicolor* (Sb). Chromosomes are depicted as circles, with curves indicating syntenic blocks of *CAD* genes; **(B)** Expression profiles of *GmCAD (FPKM)* members in roots, stems, leaves, flowers, pods, and seeds. The heatmap was generated using log_2_-transformed values, with a color gradient from green (high transcript levels) to yellow (low transcript levels).

Comparative analysis of *CAD* genes from *Arabidopsis thaliana*, *Glycine max*, *Zea mays*, *Oryza sativa*, *Phaseolus vulgaris*, and *Sorghum bicolor* provided valuable insights into the evolutionary history of *GmCADs*. As shown in **Figure 2A**, the *GmCADs* were distributed across 6 of the 20 soybean chromosomes, with 1 or 2 genes located on each of these chromosomes. A total of 12 orthologous CAD gene pairs were identified among the six species (**Supplementary Table S4**). Notably, except for the syntenic relationship between *GmCAD4* and *GmCAD7*, no synteny was detected between soybean and the other species, suggesting that the diversification of soybean CAD genes primarily occurred after species divergence. Tissue-specific expression analysis revealed distinct tissue specificity among *GmCAD*s (**Figure 2B**; **Supplementary Table S5**). *GmCAD2*, *3*, *5*, and *6* showed negligible transcript abundance across roots, stems, leaves, flowers, and seeds, whereas *GmCAD1*, *4*, and *7* were highly expressed in all examined tissues.

### 3.3 Regulatory elements in the *GmCAD* promoters

**Figure 3:**
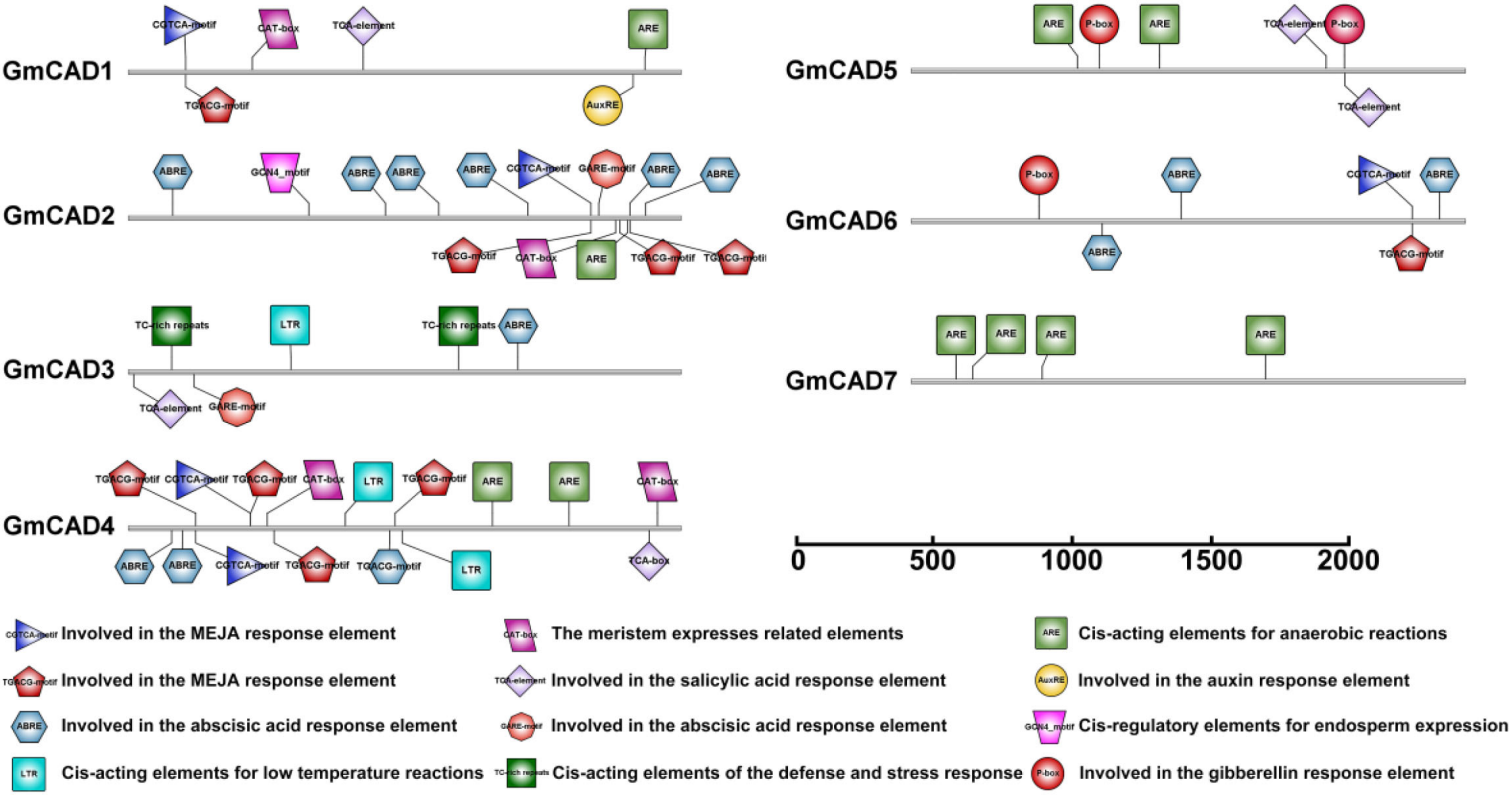
Prediction of cis-elements in the promoter regions of *GmCADs*. Boxes of different colors indicate the relative positions of the cis-elements in each *GmCAD*.

The promoter regions of each *GmCAD* gene were examined to identify potential cis-acting elements involved in transcriptional regulation. As illustrated in **Figure 3**, and **Supplementary Table S5**, most *GmCAD* genes contained hormone-responsive elements, including the auxin-responsive element (AuxRE), abscisic acid-responsive elements (ABRE and GARE-motif), gibberellin-responsive element (P-box), salicylic acid-responsive element (TCA-element), and methyl jasmonate-responsive elements (CGTCA-motif and TGACG-motif). Interestingly, while the CAT-box element (associated with meristem expression) was identified in *GmCAD1, 2*, and *4*, *GmCAD2* showed lower expression across most tissues examined, suggesting that additional regulatory mechanisms beyond the presence of this cis-element may control its tissue-specific expression pattern. Furthermore, the majority of *GmCAD* gene promoters contained stress-responsive cis-elements. Among them, the low-temperature responsive element (LTR) was present in *GmCAD3* and *4*; the anaerobic response element (ARE) was found in GmCAD1, 4, 5, and 7; and the TC-rich repeat element, involved in defense and stress responses, was identified in *GmCAD3*. These bioinformatic analyses of cis-regulatory elements suggest that *GmCAD* genes play significant roles in modulating plant stress responses and growth development.

### 3.4 Expression analysis of *GmCADs* under *Fusarium oxysporum* stress

**Figure 4:**
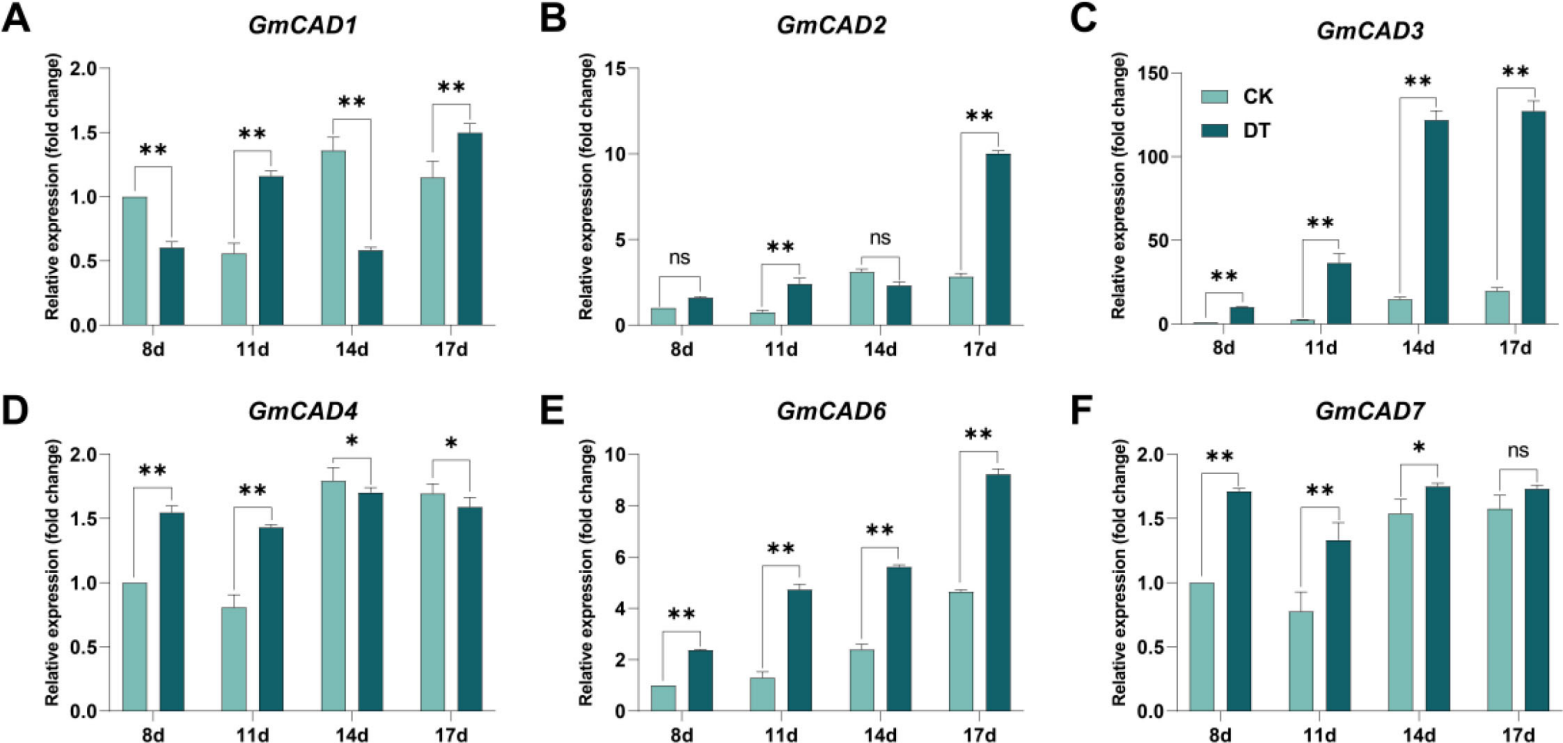
Expression analysis of *GmCAD* genes in soybean roots following *Fusarium oxysporum* inoculation. Relative expression levels of **(A)***GmCAD1*, **(B)***GmCAD2*, **(C)***GmCAD3*, **(D)***GmCAD4*, **(E)***GmCAD6*, and **(F)***GmCAD7* in roots at 8, 11, 14, and 17 days post-inoculation. CK: non-inoculated control plants (light teal bars); Fo: plants inoculated with *Fusarium oxysporum* (dark teal bars). Expression values represent mean ± SE from three biological replicates, normalized to *GmACTIN* reference gene and calculated as fold-change relative to CK at each timepoint using the 2^(-ΔΔCt) method. *GmCAD5* is not shown as no detectable expression was observed at any time point. Asterisks indicate statistically significant differences between FO and CK treatments (Student’s t-test: *P < 0.05; **P < 0.01; ns, not significant). Note: Y-axis scales differ among panels to optimize visualization of expression patterns for each gene.

To further investigate the potential role of *GmCADs* in response to *Fusarium oxysporum* stress, their expression patterns were examined at 8, 11, 14, and 17 days post-inoculation (dpi). The expression levels of all detected *GmCADs* were upregulated following pathogen challenge, except for *GmCAD5*, which showed no detectable expression. Among them, *GmCAD3* exhibited the most significant differential expression compared to the CK group. Its expression showed a time-dependent increase upon infection, peaking at 14 and 17 dpi, with levels 8.64-fold and 6.68-fold higher than those in the CK, respectively (**Figure 4**).

### 3.5 Haplotype analysis of *GmCAD3*

**Figure 5:**
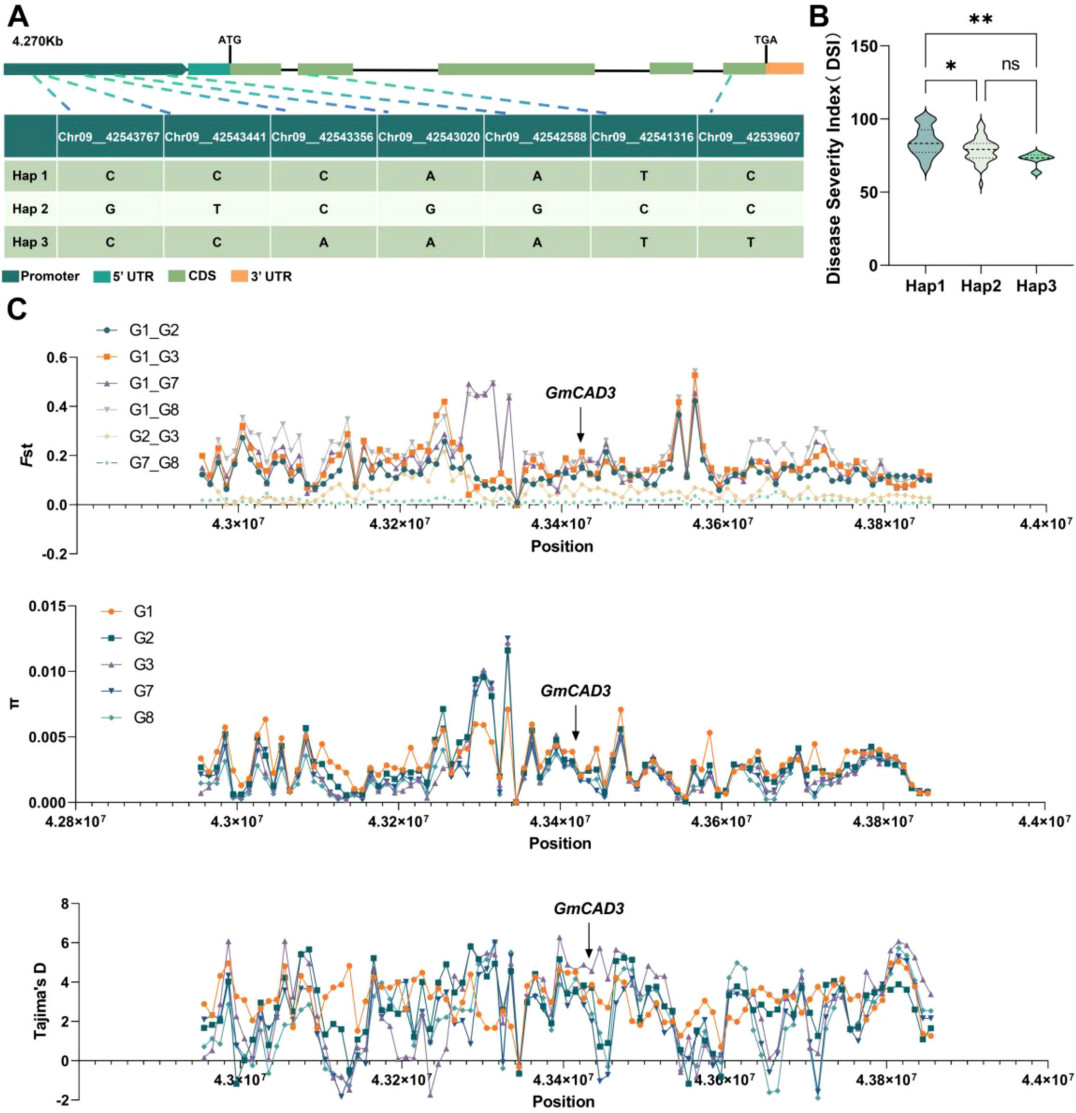
Haplotype analysis and population genetic signatures of *GmCAD3* during soybean domestication. **(A)** Gene structure of *GmCAD3* showing seven SNP positions across 4.270 kb. Colors indicate: promoter (dark green), 5’ UTR (teal), CDS (light green), and 3’ UTR (orange). ATG and TGA mark the start and stop codons, respectively. The table shows nucleotide variants for three haplotypes at five promoter SNPs (Chr09_42543767 to Chr09_42542588) and two CDS SNPs (Chr09_42541316 and Chr09_42539607). **(B)** Disease severity index (DSI) among haplotypes from 289 soybean accessions. Violin plots show DSI distribution for Hap1 (n=110), Hap2 (n=106), and Hap3 (n=73). Asterisks denote significance vs. Hap1 (Duncan’s test: *P < 0.05; **P < 0.01; ns, not significant). **(C)** Population genetics of *GmCAD3* region. Top: Fst values between wild (G1) and domesticated populations. Middle: Nucleotide diversity (π) per population. Bottom: Tajima’s D values. Arrow indicates *GmCAD3* position. Weak Fst (0.05-0.21) and positive Tajima’s D (2.36-2.97) suggest balancing selection maintaining haplotype diversity.

To investigate the association between natural variation in *GmCAD3* and disease resistance phenotypes, we performed haplotype analysis using genome resequencing data from 289 soybean accessions. Sequence analysis identified seven single-nucleotide polymorphisms (SNPs) within a 5,433 bp genomic region encompassing the *GmCAD3* gene, including five located in the 2,000 bp promoter region and two in the 1,095 bp CDS (**Figure 5A**). Based on these SNPs, the accessions were classified into three distinct haplotypes. Hap 1 was the most common (n = 110, 38.1%), while Hap 2 (n = 106, 36.7%) and Hap 3 (n = 73, 25.3%) were less frequent. Phenotypic evaluation revealed significant differences in disease resistance among haplotypes (**Figure 5B**). Accessions carrying Hap2 and Hap3 exhibited significantly lower disease indices than those carrying Hap1. Although Hap3 showed a numerically lower disease index than Hap2, this difference was not statistically significant.

### 3.6 Selective sweep analysis of *GmCAD3* during domestication

To investigate whether *GmCAD3* experienced selective pressure during soybean domestication and improvement, we analyzed population genetic parameters using genomic data from 2,790 soybean accessions retrieved from the SoyMD database (**Figure 5C**). The dataset comprised 591 wild soybeans (G1), 439 Huang-Huai-Hai landraces (G2), 379 Huang-Huai-Hai improved cultivars (G3), 539 Northeast China landraces (G7), and 842 Northeast China improved cultivars (G8). Pairwise fixation index (Fst) analysis between wild and domesticated populations revealed weak to moderate genetic differentiation (Fst = 0.05–0.21) across the *GmCAD3* genomic region, indicating the absence of strong directional selection at this locus during domestication. Notably, nucleotide diversity (π) was consistently higher in cultivated populations compared to wild accessions, a pattern contrary to expectations under strong positive selection, which typically reduces genetic variation surrounding selected alleles. Tajima’s D statistics provided further evidence against a selective sweep at the *GmCAD3* locus. Cultivated populations displayed significantly positive Tajima’s D values (ranging from 2.36 to 2.97), suggesting either balancing selection or recent population expansion rather than directional selection for a single favorable allele. These positive values indicate an excess of intermediate-frequency alleles relative to neutral expectations, consistent with the maintenance of multiple functional haplotypes.

Taken together, these population genetic signatures demonstrate that *GmCAD3* has retained substantial haplotypic diversity throughout soybean domestication and modern breeding. The persistence of multiple *GmCAD3* alleles in contemporary cultivars suggests that genetic variation at this locus provides adaptive benefits for disease resistance under diverse environmental conditions, rather than a single “optimal” allele being fixed through artificial selection.

### 3.7 Molecular and functional characterization of *GmCAD3* haplotypes

**Figure 6:**
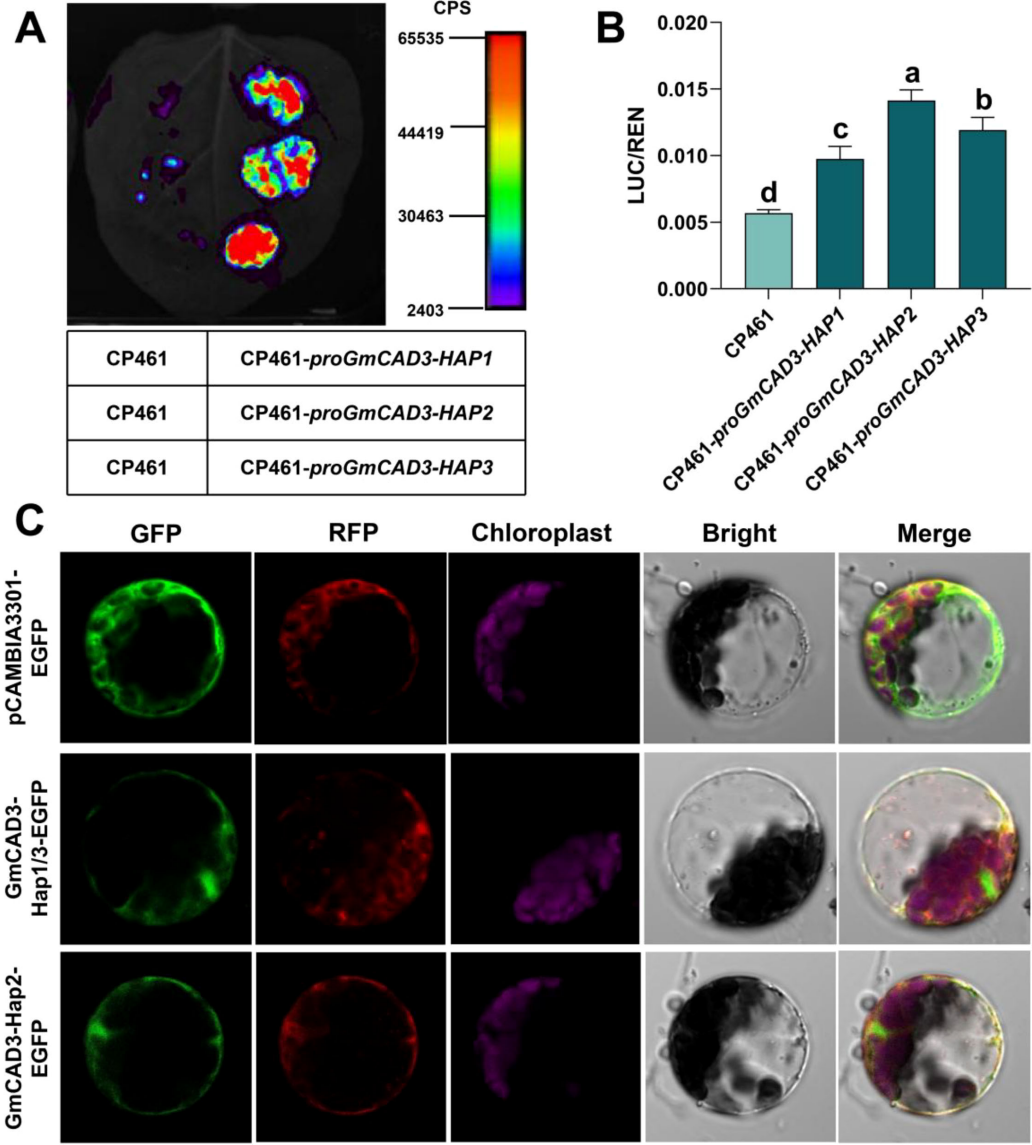
Functional Analysis of *GmCAD3* Haplotypes. **(A)** Promoter activity of different *GmCAD3* haplotypes assessed by Luc assays in *Nicotiana benthamiana* leaves. The left side of each leaf was infiltrated with the empty vector CP461 (negative control), while the right side was independently infiltrated with *proGmCAD3-Hap1*, *proGmCAD3-Hap2*, and *proGmCAD3-Hap3* constructs (top to bottom in repeated experiments). Luminescence intensity is represented by a color gradient from purple (low) to red (high). **(B)** Quantitative analysis of LUC/REN ratios for *proGmCAD3-Hap1*::Luc, *proGmCAD3-Hap2*::Luc, and *proGmCAD3-Hap3*::Luc constructs. Small letters indicate significant differences based on the ANOVA test. **(C)** Subcellular localization of *GmCAD3* haplotypes in *Arabidopsis* mesophyll protoplasts. AtHSBP-RFP was used as a cytoplasmic marker. Scale bars = 10 μm.

To investigate the functional consequences of SNPs among different *GmCAD3* haplotypes, we first analyzed their promoter sequences. Comparative analysis revealed distinct SNP profiles: *GmCAD3-HAP1* and *GmCAD3-HAP2* differed at four positions (Chr9_42543767, Chr9_42543441, Chr9_42543020, and Chr9_42542588), while *GmCAD3-HAP1* and *GmCAD3-HAP3* differed at Chr9_42543356. *GmCAD3-HAP2* and *GmCAD3-HAP3* exhibited variations at all five promoter SNP positions (**Supplementary Figure 1**; **Figure 5A**). Promoter activity was assessed using Luc assays (**Figures 6A, B**). The luminescence intensity of *proGmCAD3-HAP2*::Luc and *proGmCAD3-HAP3*::Luc was significantly stronger than that of *proGmCAD3-HAP1*::Luc. Quantitative analysis revealed that the LUC/REN ratio for GmCAD3-HAP2 (3.25 ± 0.18) was 1.18-fold and 1.44-fold higher than those of GmCAD3-HAP3 (2.76 ± 0.15) and GmCAD3-HAP1 (2.26 ± 0.12), respectively.

Analysis of translated amino acid sequences (**Supplementary Figure 2**) indicated that the SNP at Chr9_42539607 in *GmCAD3-HAP1* and *GmCAD3-HAP3* is synonymous, resulting in identical amino acid sequences. In contrast, *GmCAD3-HAP2* carries a nonsynonymous mutation at Chr9_42541316, leading to an amino acid substitution at position 46 from isoleucine (I) to valine (V). Subcellular localization analysis demonstrated that this I46V substitution did not alter protein localization, with both GmCAD3-HAP1/3 and GmCAD3-HAP2 variants localized to the cytoplasm (**Figure 6C**).

### 3.8 Functional analysis of *GmCAD3* haplotypes in soybean disease resistance

**Figure 7:**
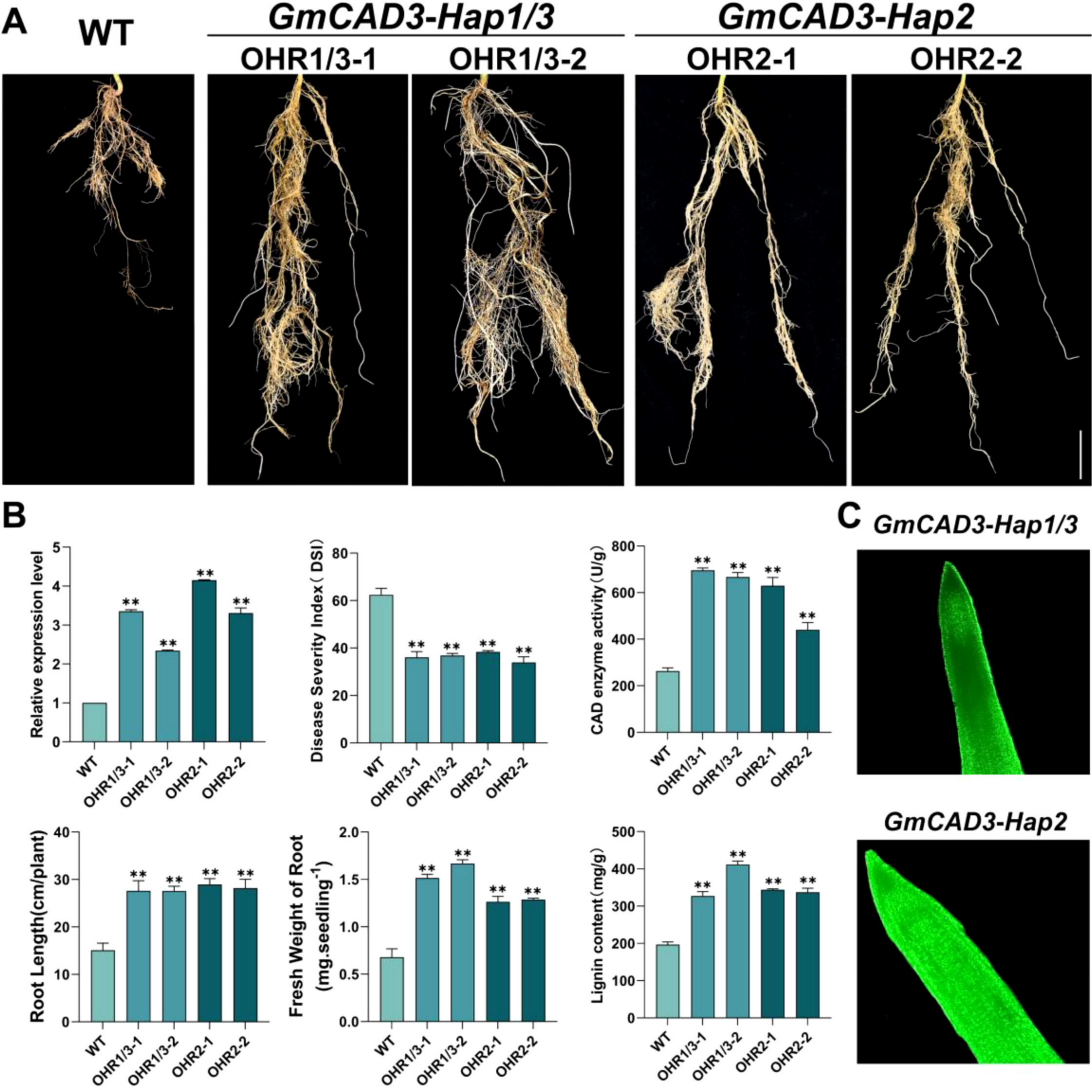
Functional validation of *GmCAD3* haplotypes in transgenic soybean hairy roots. **(A)** Phenotypes of wild-type (WT) and *GmCAD3*-overexpressing hairy root lines at 17 days post-inoculation with *Fusarium oxysporum*. Two independent transgenic lines are shown for each haplotype: OHR1/3–1 and OHR1/3-2 (*GmCAD3-Hap1/3*), OHR2–1 and OHR2-2 (*GmCAD3-Hap2*). Scale bar = 2 cm. **(B)** Quantitative analysis of transgenic lines showing: relative expression levels of *GmCAD3* (top left), disease severity index/DSI (top center), CAD enzyme activity (top right, U/g fresh weight), root length (bottom left, cm), fresh weight of root (bottom center, g per seedling), and lignin content (bottom right, mg/g fresh weight). All measurements taken at 14 days (enzyme activity and lignin content) or 17 days (other parameters) post-inoculation. Data represent mean ± SE from four independent biological replicates (n=4). Asterisks indicate significant differences compared to WT (Student’s t-test: **P < 0.01). **(C)** GFP fluorescence detection confirming transgene expression in *GmCAD3-Hap1/3* (top) and *GmCAD3-Hap2* (bottom) overexpressing hairy roots. Green fluorescence indicates successful transformation and protein expression. Images captured using confocal microscopy.

To isolate the functional effects of coding sequence polymorphisms from regulatory differences between haplotypes, the CaMV35S constitutive promoter was used to drive expression of both Hap1/3 and Hap2 coding sequences at comparable levels. This design complements our promoter activity analysis (**Figures 6A, B**), which demonstrated that the five promoter SNPs result in differential transcriptional activity among haplotypes. By standardizing expression levels through a common promoter, we could directly assess whether the nonsynonymous I46V substitution in Hap2 (**Supplementary Figure 2**) affects CAD enzyme activity or disease resistance function. To investigate the biological functions of different haplotypes of *GmCAD3* in disease resistance, transgenic soybean hairy roots overexpressing this gene were generated via *Agrobacterium rhizogenes*-mediated transformation (**Figure 7C**). qRT-PCR analysis confirmed that the transcriptional levels of *GmCAD3* in four independent overexpression lines (OHR1/3-1, OHR1/3-2, OHR2-1, OHR2-2) were 2.3 to 4.15-fold higher than those in the control wild-type (WT) hairy roots. All four independent overexpression lines were used in subsequent phenotypic, enzymatic, and biochemical analyses to ensure reproducibility of observed effects across independent transformation events. Following inoculation with *Fusarium oxysporum*, all *GmCAD3*-overexpressing hairy roots, regardless of haplotype, exhibited significantly enhanced disease resistance compared to the WT control (**Figure 7A**). Disease severity index (DSI) analysis revealed that the average DSI values of *GmCAD3-Hap1/3* and *GmCAD3-Hap2* were 36.46 and 36.11, respectively, both significantly lower than that of the WT (62.41). However, no significant difference in DSI was observed between *GmCAD3-Hap1/3* and *GmCAD3-Hap2*. Morphological trait measurements showed that *GmCAD3-Hap1/3* and *GmCAD3-Hap2* maintained longer root lengths (27.61 cm and 28.57 cm, respectively) and higher root fresh weights (1.59 g and 1.28 g, respectively), which were significantly greater than those of the WT (15.08 cm, 0.68 g; **Figure 7B**). No significant morphological differences were detected between the two haplotypes.

To further examine whether *GmCAD3* enhances disease resistance in soybean through CAD enzyme activity and lignin accumulation, we measured both CAD activity and lignin content in the four overexpression lines. As shown in **Figure 7B**, CAD activities in *GmCAD3-Hap1/3* and *GmCAD3-Hap2* were 695.90 U/g and 535.00 U/g, respectively, significantly higher than that in the WT (263.18 U/g). Similarly, after inoculation with *F. oxysporum*, lignin content in the overexpression hairy roots of *GmCAD3-Hap1/3* and *GmCAD3-Hap2* increased significantly to 369.51 mg/g and 340.62 mg/g, respectively, compared to 196.68 mg/g in the WT (**Figure 7B**). These results indicate that *GmCAD3* enhances resistance to pathogen infection by elevating CAD activity and promoting lignin accumulation, thereby alleviating root damage. Furthermore, the SNP variations in the coding sequence among different *GmCAD3* haplotypes did not lead to significant functional differences.

## 4 Discussion

CAD is a naturally occurring enzyme that plays a vital role in plant development and growth, especially in the plant’s response to biotic stress (Rajput et al., 2021; Jiang et al., 2022). Research on CAD genes has primarily focused on other species to date. Various plants, including wheat (Rong et al., 2016), cotton (Li et al., 2022), and Arabidopsis (Kim et al., 2004). However, there is limited information about the CAD gene family in soybean. Seven CAD genes have been identified in the soybean genome and are named *GmCAD1-7*. The *GmCAD* proteins encoded by these genes contain the same essential and distinct protein domains (PF08240 and PF00107) as other typical CADs (Li et al., 2022). These conserved domains are responsible for CAD catalytic activity and indicate the functional conservation of these enzymes across plant species.

### 4.1 Analysis of molecular characteristics of the *GmCAD* family

The CAD family has undergone species-specific expansion throughout evolution, as demonstrated by comparing CAD families from various plant species (Zhao et al., 2020; Zhang et al., 2021; Li et al., 2022). Recently, new functions of CAD have been reported in wheat (Rong et al., 2016), cotton (Li et al., 2022), and Arabidopsis (Li et al., 2022), including roles in lignin biosynthesis, cell wall formation, and disease resistance. Tissue-specific expression analysis revealed distinct patterns among *GmCAD* members. *GmCAD1*, *4*, and *7* were found to have high expression levels across all examined tissues, whereas *GmCAD2*, *3, 5*, and *6* showed negligible transcript abundance in roots, stems, leaves, flowers, and seeds. The numerous potential physiological roles of *GmCADs* in soybean development are supported by their expression patterns in various tissues. The transcriptional responses of *GmCADs* to *Fusarium oxysporum* infection were examined, showing significant upregulation of *GmCADs* under pathogen attack, especially *GmCAD3.* Likewise, *TaCAD12* (Rong et al., 2016), and *GhCAD7* (Tuerxun et al., 2025), transcripts are significantly upregulated in response to pathogen infection and play crucial roles in disease resistance. Cis-regulatory elements in the promoter regions may mediate transcriptional activation of CAD genes in response to biotic stress (Ijaz et al., 2020; Xu et al., 2024). Most cis-acting elements in *GmCAD* promoters likely regulate stress and hormone responses, including ABRE, GARE-motif, TCA-element, CGTCA-motif, and TGACG-motif, as well as stress-response elements such as LTR, TC-rich repeats, and ARE. Phylogenetic analysis shows *that GmCADs* form four groups, mainly in Cluster 2 with common bean and *AtCAD6, suggesting* conserved functions in legumes. Synteny analysis reveals limited cross-species relationships, except for *GmCAD4*-*GmCAD7*, suggesting soybean CAD gene diversification occurred mainly after species divergence. This pattern differs from many other gene families that show widespread synteny across plant lineages, implying that lineage-specific selection pressures may shape CAD genes (Zhao et al., 2017; Wang et al., 2025).

### 4.2 *GmCAD3* boosts disease resistance by increasing lignin via CAD enzyme activity, enhancing soybean’s *Fusarium oxysporum* tolerance

*GmCAD3* responded more rapidly and strongly to *Fusarium oxysporum* than other genes, with maximal induction (~8.64-fold) at 14 days post-inoculation, indicating a key role in pathogen stress response. Using the hairy root system, it was confirmed that GmCAD3 encodes a cinnamyl alcohol dehydrogenase. Overexpressing *GmCAD3* in soybean hairy roots increased root length and weight, suggesting improved disease tolerance. These results align with earlier studies, where overexpressing CAD genes markedly enhanced disease resistance in wheat and other plant species (Rong et al., 2016; Zhang et al., 2022; Zhou et al., 2025a, b).

Cell wall reinforcement through lignin deposition is a crucial defense mechanism under pathogen attack (Gao et al., 2024; Uddin et al., 2024). In pathogen-challenged situations, CAD is essential for the production of lignin monomers (Zhang et al., 2022). During *Fusarium oxysporum* infection, the CAD enzyme activity in *GmCAD3-OHR* increased considerably, and a significant accumulation of lignin was observed. Consistent with earlier studies, our observations confirmed the role of *GmCAD3* in enhancing disease resistance by catalyzing the final step in monolignol biosynthesis and promoting lignin accumulation (Roychowdhury et al., 2025; Wang et al., 2025). Recent evidence indicates that lignin functions as a physical barrier that strengthens cell walls and limits pathogen invasion (Zhou et al., 2025b). The temporal expression pattern of *GmCAD3*, which shows progressive induction over time and peaks at 14–17 days post-inoculation, aligns with the establishment of defense lignification, which requires sustained gene expression and metabolite accumulation. In contrast, constitutively expressed *GmCAD* members (*GmCAD1, 4*, and *7*) are likely to contribute to developmental lignification rather than to stress-induced responses.

### 4.3 Variation in the *GmCAD3* gene is closely related to the disease resistance of soybean

The widespread application of whole-genome sequencing-based haplotype analysis in crops has been used in recent years to mine beneficial alleles from natural variability, narrow the target range, and improve the accuracy of target gene identification (Yang et al., 2023). This approach has been increasingly employed to assess candidate genes and study the role of natural variation in disease resistance traits (Sang et al., 2023). The *GmCAD3* gene harbored 7 significant SNPs, 5 in the promoter region and 2 in the coding sequence. Three haplotypes were identified in 289 soybean accessions: Hap2 and Hap3 showed lower disease severity than Hap1, indicating that variants confer resistance to *Fusarium oxysporum*. Promoter analysis showed that these SNPs differentially affected transcriptional activity, with pro*GmCAD3*-HAP2:Luc exhibiting the strongest luminescence, followed by hap3 and hap1. The LUC/REN ratio was 1.18 and 1.44 times higher for HAP2 than for HAP3 and HAP1, respectively, indicating that promoter polymorphisms affect gene expression, likely by altering transcription factor binding sites, thereby influencing *GmCAD3* transcription, lignin levels, and disease resistance.

The two SNPs in the coding sequence differentiated GmCAD3-HAP2 from the other haplotypes. Analysis revealed that one SNP represents a synonymous mutation in GmCAD3-HAP1 and GmCAD3-HAP3, while the other causes a nonsynonymous mutation in GmCAD3-HAP2, resulting in an amino acid substitution from isoleucine to valine at position 46. Subcellular localization analysis demonstrated that this substitution did not alter protein localization, with both variants remaining in the cytoplasm.

To determine whether this I46V substitution affects protein function, we conducted functional validation through hairy root overexpression using a constitutive CaMV35S promoter. This experimental design was specifically chosen to isolate coding sequence effects from the regulatory differences already characterized through promoter activity analysis (**Figures 6A, B**). The results showed that both GmCAD3-Hap1/3 and GmCAD3-Hap2 exhibited comparable disease resistance (DSI: 36.46 vs 36.11), CAD enzyme activity (695.90 vs 535.00 U/g FW), and lignin content (369.51 vs 340.62 mg/g DW) when expressed at similar levels (**Figure 7B**). These results indicate that the I46V substitution, involving two hydrophobic amino acids with similar chemical properties, does not significantly affect protein catalytic function or disease resistance capacity.

Integrating our findings across multiple experimental approaches reveals a clear mechanistic picture: the differential disease resistance observed among GmCAD3 haplotypes in natural germplasm accessions (**Figure 5B**) is primarily driven by promoter variation affecting expression levels rather than by coding sequence changes affecting protein function. The promoter activity analysis showed that HAP2 exhibits 1.18-fold and 1.44-fold higher transcriptional activity than HAP3 and HAP1, respectively (**Figure 6B**), while the protein functional validation demonstrated equivalent enzymatic capacity across haplotypes. This finding has important implications for molecular breeding strategies: marker-assisted selection should prioritize favorable promoter haplotypes that enhance GmCAD3 expression, as the protein variants themselves are functionally equivalent. The higher promoter activity of HAP2, combined with its significantly lower disease severity index in germplasm evaluation, supports this variant as the optimal target for improving Fusarium resistance in soybean breeding programs.

## 5 Conclusions

This study presents the first comprehensive genome-wide identification and functional analysis of the CAD gene family in soybean. We identified seven *GmCAD* genes with distinct evolutionary backgrounds, expression patterns, and regulatory features. Among these, *GmCAD3* stood out as a key regulator of disease resistance to *Fusarium oxysporum*, acting by enhancing CAD enzyme activity and increasing lignin accumulation. The analysis of three natural *GmCAD3* haplotypes, including Hap2, which shows evidence of favorable promoter activity, provides valuable molecular markers for breeding disease-resistant soybean cultivars. The persistence of multiple functional haplotypes in modern germplasm, supported by population-genetic evidence of balancing selection, underscores the adaptive importance of genetic diversity at this locus. These findings deepen our understanding of lignin-driven plant immunity and provide practical tools for sustainable disease management in soybean cultivation. Combining marker-assisted selection of favorable *GmCAD3* alleles with other resistance breeding strategies holds promise for combating the rising threat of Fusarium root rot amid changing climate conditions and intensive farming practices.

## Data Availability

The datasets presented in this study can be found in online repositories. The names of the repository/repositories and accession number(s) can be found in the article/**Supplementary Material**.
