# Supplementary figures and images for: Genome-wide analysis of soybean cinnamyl alcohol dehydrogenase genes identifies GmCAD3 as a positive regulator of Fusarium oxysporum resistance
Source: Front Plant Sci. 2025 Dec 11;16:1731612. doi: 10.3389/fpls.2025.1731612 (PMC12738375; doi:10.3389/fpls.2025.1731612)

**
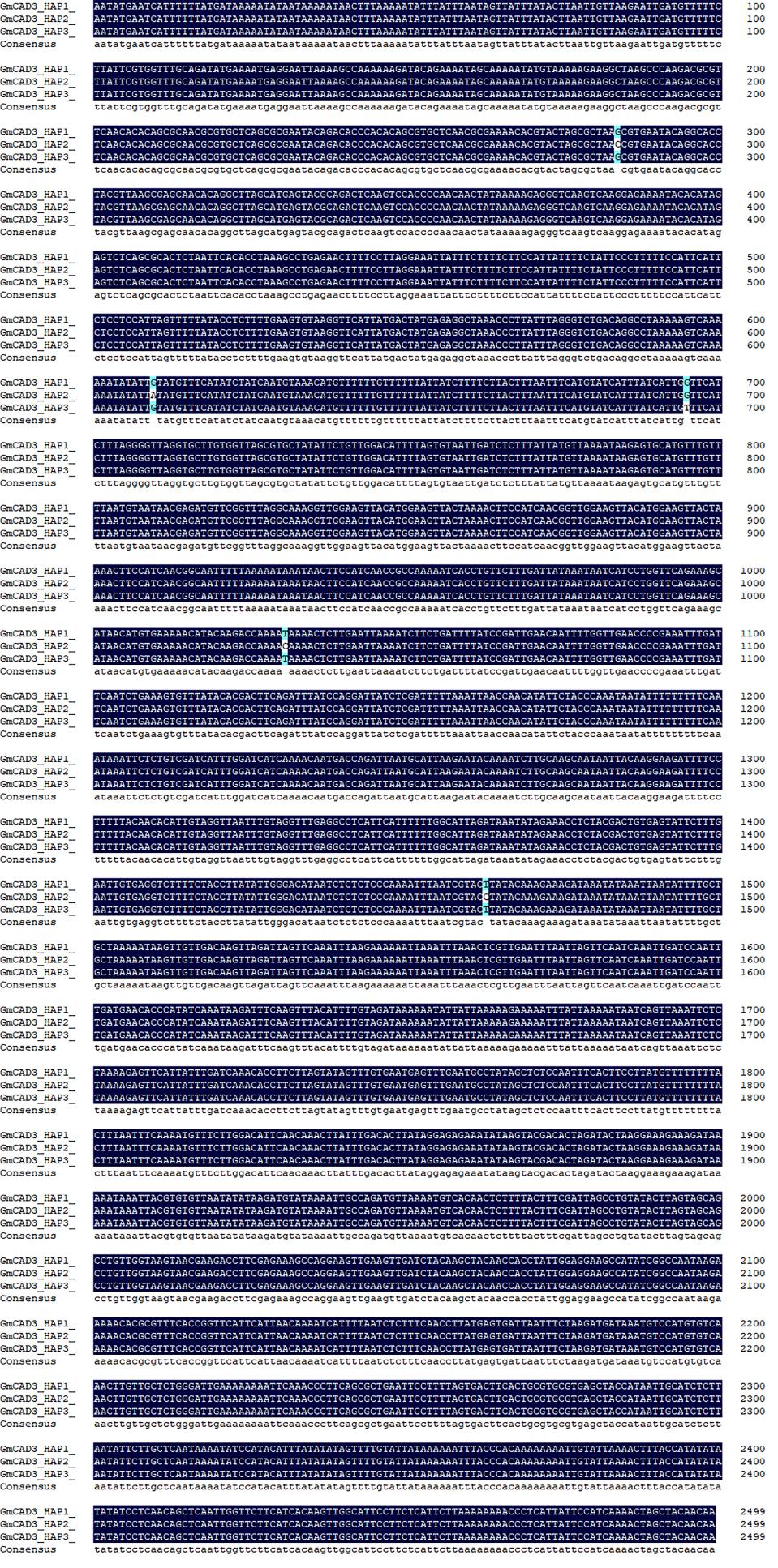
**

**Supplementary Figure 1** Comparison of *GmCAD3-Hap1*, *GmCAD3-Hap2*, and *GmCAD3-Hap3* promoter sequences

Supplement: Supplementary file 1 [file DataSheet1.docx]

**
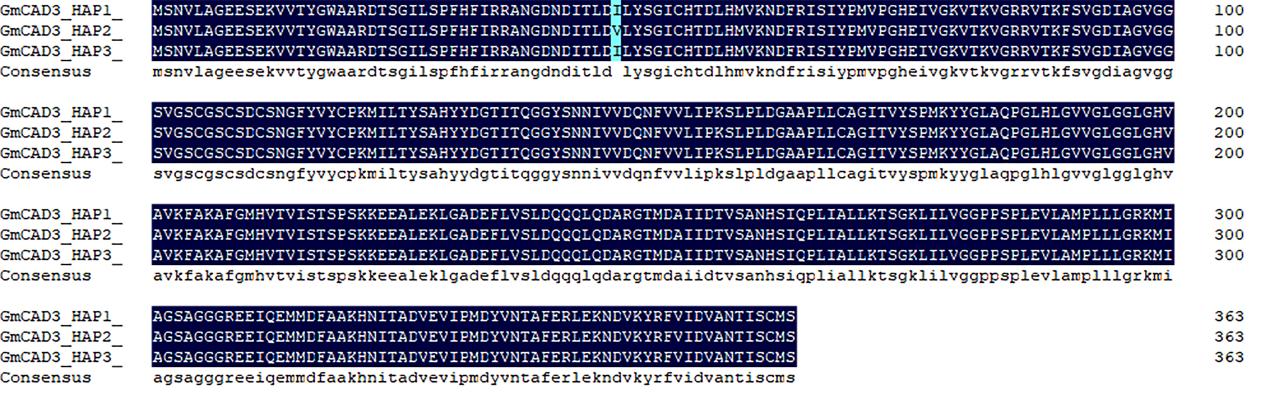
**

**Supplementary Figure 2** Comparison of GmCAD3-Hap1, GmCAD3-Hap2, and GmCAD3-Hap3 amino acid sequences

Supplement: Supplementary file 2 [file DataSheet2.docx]
